# Locally Advanced Adrenocortical Carcinoma in Children and Adolescents—Enigmatic and Challenging Cases

**DOI:** 10.3390/cancers15174296

**Published:** 2023-08-28

**Authors:** Michaela Kuhlen, Pascal Mier, Marina Kunstreich, Lienhard Lessel, Christoph Slavetinsky, Jörg Fuchs, Guido Seitz, Paul-Martin Holterhus, Stefan A. Wudy, Christian Vokuhl, Michael C. Frühwald, Peter Vorwerk, Antje Redlich

**Affiliations:** 1 Pediatrics and Adolescents Medicine, Faculty of Medicine, University of Augsburg, 86156 Augsburg, Germany; 2 Pediatric Hematology/Oncology, Department of Pediatrics, Otto von Guericke University Children’s Hospital, 39106 Magdeburg, Germany; 3 Department of Paediatric Surgery and Paediatric Urology, University Children’s Hospital Tuebingen, 72076 Tuebingen, Germany; 4 Department of Pediatric Surgery and Urology, University Hospital Giessen-Marburg, 35043 Marburg, Germany; 5 Division of Paediatric Endocrinology and Diabetes, Department of Paediatrics, University Hospital Schleswig-Holstein, Christian-Albrechts-University Kiel, 24105 Kiel, Germany; 6 Paediatric Endocrinology & Diabetology, Steroid Research & Mass Spectrometry Unit, Centre of Child and Adolescent Medicine, Justus Liebig University, 35392 Giessen, Germany; 7 Section of Pediatric Pathology, Institute of Pathology, University of Bonn, 53127 Bonn, Germany

**Keywords:** adrenocortical carcinoma, children and adolescents, locally advanced disease

## Abstract

**Simple Summary:**

Pediatric COG stage II and III adrenocortical carcinoma (ACC) is one of the most unpredictable and challenging tumors in terms of prognosis and management. The EXPeRT/PARTNER recommendation suggested inclusion of the five-item microscopic score to guide management in these patients. In this study, we report on 18 pediatric ACC stage II and 37 stage III patients registered with the German MET studies. We explored the five-item microscopic and the pS-GRAS score to predict outcome. Three-year event-free survival estimates were 76.5% in stage II and 49.8% in stage III patients. In COG stage III patients, EFS was impaired for patients with unfavorable histology according to the five-item score. No differences were observed in stage II patients and in the pS-GRAS groups. Further research including molecular studies is needed to identify high-risk features in locally advanced pediatric ACC tumors.

**Abstract:**

Background: Locally advanced tumors account for approximately 50% of children and adolescents with adrenocortical carcinoma (ACC), and of these, up to 50% relapse. We explored the five-item microscopic score and the pS-GRAS score for guiding management. Methods: Data from children and adolescents with COG stage II and III ACC registered in the MET studies were included. The five-item and pS-GRAS score were retrospectively calculated. Results: By December 2021, 55 patients with stage II and III (stage II *n* = 18, stage III *n* = 37) had been reported. Median age was 4.3 years [0.1–17.8], median duration of follow-up 6.0 years [0–16.7]. 3-year event-free survival (EFS) rate was 76.5% and 49.8% (*p* = 0.088), respectively. In stage II tumors, neither the five-item score (*p* = 0.872) nor pS-GRAS grouping (*p* = 0.218) had any effect as prognostic factors. In stage III patients, EFS was impaired in tumors with unfavorable histology according to the five-item score (100% vs. 30.8%, *p* = 0.018). No difference was observed for pS-GRAS groups (*p* = 0.798). Conclusions: In patients with COG stage III, but not stage II, the five-item score affected EFS. Further studies are needed to identify patients at risk in COG stage II.

## 1. Introduction

Adrenocortical carcinoma (ACC) is a rare endocrine malignancy with an aggressive clinical course and high mortality rate in children and adolescents [1,2]. Surgery is the mainstay of therapy [1,3,4]. For children and adolescents with advanced disease, mitotane, an adrenolytic drug, has been suggested with or without chemotherapy [1,3].

Approximately 40–50% of children and adolescents with ACC are diagnosed with locally advanced disease [3,5,6]. Up to 50% of them relapse after complete tumor resection [3,4,5,6,7,8,9]. We have previously reported that only a small proportion of these patients can be rescued by salvage therapy [10].

The goal of complete tumor resection in patients with ACC is impeded by the fragility of the tumor and its invasiveness into adjacent structures [11]. In addition, the increased risk of recurrence in children and adolescents with locally advanced disease is poorly understood. Data from adults suggested that residual tumor in retroperitoneal lymph nodes may contribute to recurrence [12,13].

Therefore, in the prospective single-arm risk-stratified interventional study ARAR0332 of the Children’s Oncology Group (COG), COG stage II patients were treated with surgery and retroperitoneal lymph node dissection [3]. Event-free survival (EFS) was 53.5% and thus, could not be improved. In contrast, EFS increased to 81% in COG stage III patients treated with mitotane and CED (cisplatin, etoposide, doxorubicin) chemotherapy. However, this combination therapy resulted in significant toxicity. According to the study regulations and the authors’ conclusions, the regimen was not feasible, and modifications would be required to improve tolerability. Of note, a substantial number of patients with stage I and stage II tumors were included in this risk group because of tumor spillage. This may have influenced the better EFS.

Since our understanding of the risk of recurrence and thus of the optimal treatment in children and adolescents with locally advanced disease is still incomplete, it can be assumed that a considerable number of these patients are under- or over-treated and accordingly suffer the resulting far-reaching consequences.

Based on a national cohort study, Picard and colleagues proposed a two-tier pathologic approach with a five-item score for COG stage II and III patients to determine the need of adjuvant systemic therapy [11]. This two-tier approach was subsequently adopted in the diagnostic and therapeutic recommendations for adrenocortical tumors in children and adolescents of the European Cooperative Study Group for Pediatric Rare Tumors (EXPeRT) [1]. Recently, Riedmeier and colleagues published a modified (pediatric) S-GRAS score adapted from the European Network for the Study of Adrenal Tumors for prognostic classification of adult ACC [14,15].

Here, our goal is to improve our understanding of locally advanced ACC. To this end, we investigate the clinical characteristics, management, and outcomes in patients with COG stage II and III registered with the German Malignant Endocrine Tumor (MET) studies. We examine the five-item microscopic score proposed by Picard et al. [1] and the pediatric S-GRAS score [14] for guiding adjuvant treatment. We discuss the implications of our findings for clinical strategies and future research.

## 2. Materials and Methods

Children and adolescents aged <18 years with adrenocortical tumors were prospectively registered in the national multicenter MET studies between January 1997 and December 2021. We analyzed patients with histologically confirmed diagnosis of ACC. We retrospectively assigned the post-surgical tumor status according to the COG staging system [1,7,16]. Patients with COG stage II and III were included in this analysis. Follow-up for this study was completed on 31 December 2022.

The GPOH-MET 97 protocol and the GPOH-MET 2013 registry were approved by the ethics committees of the University of Luebeck (Approval number 97125) and the Otto-von-Guericke-University Magdeburg (Approval number 174/12), Germany. Informed consent was obtained from patients, parents, and legal guardians, as appropriate.

### 2.1. The MET Studies

Patients were treated according to the GPOH-MET 97 study protocol and registry recommendation. Briefly, for patients with completely resected stage I and II (T1-2, N0, M0) tumors and T3, N0, M0 stage III tumors (AJCC 7th edition staging system) no additional treatment was recommended. For patients with T1-2, N1, M0 and incompletely resected T3, N0, M0 stage III tumors, four cycles of chemotherapy (alternating NN-1: vincristine, ifosfamide, doxorubicin; and NN-2: carboplatin and etoposide) with mitotane over a nine-month period was suggested. Two to four cycles of neoadjuvant chemotherapy with mitotane were recommended for patients with primarily unresectable tumors.

T stage, tumor volume, lymph node involvement, Ki67 index, mitoses, and local invasion and invasion into surrounding tissue were extracted from the histopathological reports in the case of tumor resection. In case of inoperability, tumor volumes and lymph node involvement were estimated from radiological images. We defined steroid profiles as suspicious if the plasma and/or urine steroid hormone levels were elevated according to local reference ranges. Stage of puberty was defined as ‘prepubertal’ in females < 8 years of age and in males < 9 years of age and ‘pubertal’ in all other cases.

Tumors were retrospectively classified into ‘favorable’ and ‘unfavorable’ histology according to the 5-item microscopic score by Picard et al. [11] and into group 1 to 4 according to the pediatric scoring system pS-GRAS proposed by Riedmeier et al. [14] (Supplementary Figure S1).

Recurrence was defined as new hormonal or structural evidence of disease following any period of complete remission (CR), that is, the absence of any functional or structural evidence of disease. Disease progression (PD) was defined as any new evidence of disease without prior CR.

### 2.2. Statistical Analyses

Event-free survival (EFS) was defined as time from diagnosis to the earliest time of disease progression, relapse, or death from disease, and was estimated using the method of Kaplan and Meier. Times for living patients were censored at the last follow-up.

Groups were compared using the log-rank test, chi-squared test, *t*-test, and Mann–Whitney U test. For all exploratory testing, a *p* value ≤ 0.05 was considered statistically significant.

Statistical analyses were performed using SPSS version 26. Data visualization and graphs were created using SPSS and R version 4.0.5 using the packages ‘ggplot2′, ‘survial’ and ‘survminer’.

## 3. Results

**Figure 1:**
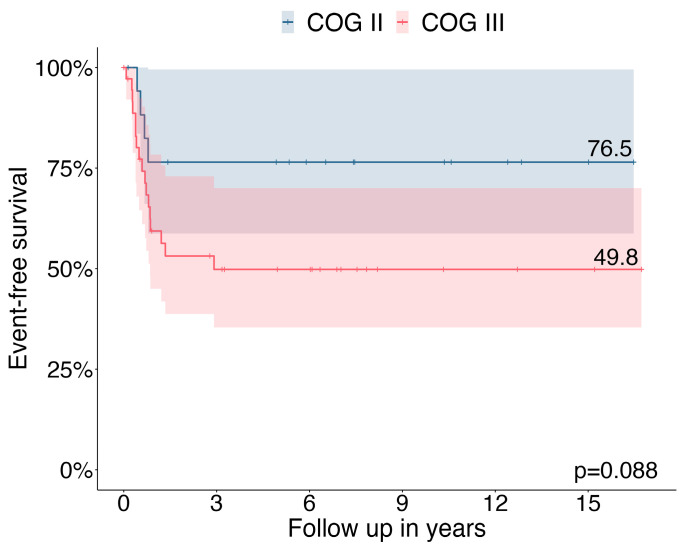
Three-year event free survival in patients with COG stage II (*n* = 18) and stage III (*n* = 37).

In the MET database, 55 children and adolescents with locally advanced ACC [COG stage II *n* = 18 (32.7%) and COG stage III *n* = 37 (67.3%)] were registered with a median age at diagnosis of 4.3 years (range, 0.1–17.8 years). The median duration of follow-up was 6.0 years (with a range of 0–16.7). The 3-year EFS was 76.5% in COG stage II patients and 49.8% in COG stage III patients (Figure 1).

### 3.1. COG Stage II Patients

Of 18 patients with COG stage II, 14 (77.8%) were female. The steroid profile was abnormal in 18 (100%) patients. The right and left adrenal glands were affected in 10 (55.6%) and 7 (38.9%) patients, respectively, and one patient had bilateral tumors. The clinical characteristics and histopathologic details of patients with COG stage II are shown in Table 1.

Adrenalectomy was performed in 17 patients and combined adrenalectomy and nephrectomy in one patient. This was complemented by thrombectomy in three patients because of a tumor in the renal vein (*n* = 1) or because of a tumor in the inferior vena cava (*n* = 2), including reconstruction of the vena cava in one case.

Microscopic complete tumor resection was reported in 18 (100%) patients, and spillage in none. In 10 (of 17; 58.8%) patients, histopathologic examination demonstrated preexisting invasion into or perforation of the tumor capsule. In another patient, it was lesioned during surgery. Microscopic vascular invasion was detected in 13 (of 16; 81.3%) patients. Venous invasion was limited to local veins in 4 (of 18; 22.2%) patients, comprised the renal vein in 1 (5.6%) patient and invading the vena cava in 3 (16.7%) patients. According to the local pathologist’s assessment, 9 (of 17; 52.9%) patients were diagnosed with stage pT2, 1 (5.9%) patient with pT3, and 7 (41.2%) patients with pT4. No patient was diagnosed with pT1.

Tumor necrosis was reported in 14 (of 16; 87.5%) patients and Ki67 index > 15% in 8 (of 17; 47.1%) patients.

In 10 (of 18; 55.6%) patients, at least one lymph node was removed during surgery (median lymph nodes examined: 2, range 1–20) with no histopathologic lymph node involvement detected in any patient.

In 3 (of 18; 16.7%) patients, neoadjuvant treatment was administered, 4 (22.2%) patients underwent adjuvant chemotherapy including mitotane therapy in 3 (16.7%) patients.

#### Events in COG Stage II Patients

Four (of 18; 22.2%) patients relapsed after microscopically complete tumor resection. Among these patients, one patient was diagnosed with a tumor volume of 3645 mL and unfavorable histology according to the 5-item score, pS-GRAS score group 4. This patient was diagnosed with new lung metastases following 4 cycles of chemotherapy and while on ongoing mitotane therapy. The second patient was found to have a tumor volume of 267 mL and unfavorable histology, pS-GRAS group 4. Five months later, the patient was diagnosed with metastases in the liver and ipsilateral kidney. The third patient was found to have a tumor volume of 115 mL and unfavorable histology, pS-GRAS group 3. Six months later, this patient was diagnosed with multiple metastases in the liver and lung. The fourth patient had missing data for calculation of the 5-item score. He had a tumor volume of 150 mL, a Ki67 index < 15%, and pS-GRAS group 3. Eight months later, this patient was diagnosed with a solitary liver metastasis.

Patients with event were significantly older than patients without (7.7 years vs. 2.4 years, *p* = 0.047). No differences were observed between patients with and without events with respect to the endocrine phenotype (*p* = 0.170), laterality (*p* = 0.641), T stage (*p* = 0.571), tumor volume (*p* = 0.491), adrenal capsular invasion (*p* = 1.000), venous invasion (*p* = 1.000), Ki67 index > 15% (*p* = 0.479), 5-item score (*p* = 0.872), and pS-GRAS group (*p* = 0.218) as well as adjuvant chemotherapy (*p* = 1.000) and mitotane therapy (*p* = 1.000).

### 3.2. COG Stage III Patients

Of 37 patients with COG stage III, 14 (37.8%) were female. The steroid profile was suspicious in 33 (of 35; 94.3%) patients. Slightly more tumors occurred in the right adrenal gland (right to left ratio 1.2:1). The clinical and pathological details of patients with COG stage III are shown in Table 2.

Of 36 patients, 21 (58.3%) patients underwent adrenalectomy, which was combined with nephrectomy in 2 (5.6%) and lymph node resection in 8 (22.2%) additional patients. A complex surgical approach including the abdominal and thoracic cavities was reported in 4 (11.1%) patients. No detailed information on the surgical approach was provided in 1 patient.

Microscopically complete tumor resection was demonstrated in 21 (of 35; 60.0%) patients, microscopic and macroscopic residues were demonstrated in 10 (28.6%) and 4 (11.4%) patients; microscopic and macroscopic remnants were detected in 10 (28.6%) and 4 (11.4%) patients, respectively. Tumor spillage was reported in 16 (of 37; 43.2%) patients. Pre-existing invasion into or perforation of the tumor capsule was determined by histopathological evaluation in 8 (of 18; 44.4%) patients and vascular invasion in 18 (of 28; 64.3%) patients. According to radiological evaluation, adjacent organs were affected in 8 (of 34; 23.5%) patients (great vessels *n* = 6, liver *n* = 1).

Of 34 patients, 2 (5.9%) were classified as pT1, 20 (58.8%) as pT2, 1 (2.9%) patient as pT3, and 11 (32.3%) as pT4.

Tumor necrosis was present in 28 (of 34; 65.1%) patients and Ki67 index > 15% in 23 (of 31; 74.2%) patients.

In 36 patients, N stage was reported including 32 (88.9%) patients with stage N0 and 4 (11.1%) patients with stage N1.

Neoadjuvant therapy was administered in 6 (of 37; 16.2%) patients, 19 (of 35; 54.3%) patients underwent adjuvant chemotherapy including mitotane therapy in 17 patients.

#### Events in COG Stage III Patients and Prognostic Factors (Univariate Analysis)

Of 37 patients, 1 (2.7%) patient was diagnosed with progressive disease, 16 (43.3%) patients with relapse (locoregional including the abdominal cavity *n* = 9, metastatic *n* = 5, combined *n* = 2). Unfavorable histology was detected in 11 patients (incomplete data in 6 patients) and pS-GRAS group 2, 3, and 4 were assigned in 1, 5, and 8 patients, respectively (incomplete data in 3 patients).

In patients with event compared to patients without event, Ki67 index > 15% (*p* = 0.018) and unfavorable histology (*p* = 0.017) were significantly more frequent, and tumor volume was significantly higher (*p* = 0.017). ROC curve analysis demonstrated that tumor volume > 300 mL was best for classifying patients in COG stage III with and without subsequent events (sensitivity 76.5%, specificity 75.0%).

No differences were observed for patients with and without events with respect to age (*p* = 0.968), endocrine phenotype (*p* = 0.692), laterality (*p* = 0.648), T stage (*p* = 0.113), adrenal capsular invasion (*p* = 0.177), venous invasion (*p* = 0.366), invasion into adjacent organs (*p* = 0.402), N stage (*p* = 0.441), resection status (*p* = 0.991), tumor spillage (*p* = 0.444), and pS-GRAS group (*p* = 0.819) as well as adjuvant chemotherapy (*p* = 1.000) and mitotane therapy (*p* = 1.000).

**Figure 2:**
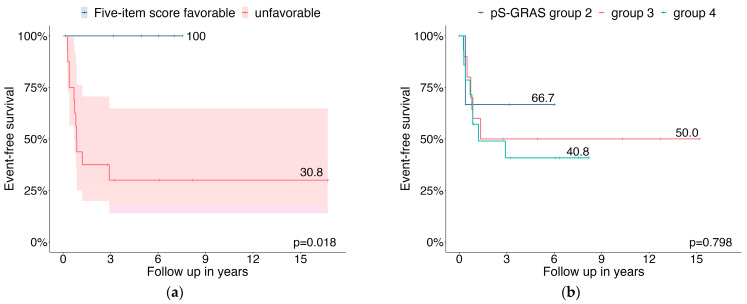
Probabilities of event-free survival for COG stage III patients relating to (**a**) 5-item microscopic score; (**b**) pS-GRAS group.

Differences in EFS were observed between patients with favorable and unfavorable histology according to the 5-item score (100% vs. 30.8%, *p* = 0.018) (Figure 2a). The pS-GRAS groups had no significant effect on EFS as a prognostic factor (group 2: 66.7% vs. group 3: 50.0% vs. group 4: 40.8%, *p* = 0.798) (Figure 2b).

## 4. Discussion

Consistent with previous reports, our data confirm that locally advanced ACC in children and adolescents is associated with a dismal prognosis (3-year EFS 76.5% in COG stage II and 49.8% in COG stage III). In COG stage II patients, neither the five-item microscopic score [11] nor pS-GRAS grouping [14] significantly affected EFS as prognostic factors. In contrast, in patients with stage III COG, unfavorable histology according to the five-item score significantly affected EFS as a negative prognostic factor. No significant differences were observed between pS-GRAS group 2, 3, and 4 in terms of EFS.

Approximately 50% of children and adolescents with ACC are diagnosed with locally advanced disease. Management of these patients is nowadays guided based on COG staging [1,3]. Many patients suffer from significant toxicity in the setting of adjuvant systemic treatment [3]. Up to 50% of stage II-III patients still experience disease recurrence [3,5,6,7]. Given these data, a more sophisticated risk stratification is needed to guide adjuvant treatment.

To address this shortcoming, Picard and colleagues proposed a two-step approach including COG staging and a five-item microscopic score, which was independently associated with prognosis in the French cohort [11]. We recently reported that an unfavorable five-item score had a significant impact on EFS and OS in a multivariate Cox regression model in children and adolescents with ACC enrolled in the German MET studies [5]. The EXPeRT/PARTNER recommendations adopted this approach and suggested considering adjuvant therapy in COG stage II-III patients in case of unfavorable histology.

We investigated this approach in children and adolescents with ACC registered with the German MET studies. In 18 COG stage II patients, those with events were significantly older than without events. We observed no difference between patients with favorable and unfavorable tumor histology with respect to subsequent events. Though, in 37 COG stage III patients, EFS was worse in patients with unfavorable histology. In the study by Picard and colleagues, no significant differences between patients with favorable and unfavorable histology for stage II and stage III patients were observed [11]. The authors acknowledged the insufficient number of patients for the subgroup analysis as major limitation of the study. Indeed, only 15 patients each with COG stage II and stage III were included in this study. This is comparable to the number of stage II patients in our study, whereas we included more patients with stage III. In line with the study by Picard and colleagues, no deaths occurred when the five-item microscopic score was <2 regardless of stage. However, in our study, 8 stage II and 5 stage III patients with unfavorable histology remained in first complete remission (undergoing adjuvant therapy in some cases) calling into question the prognostic specificity of the five-item score in unfavorable histology. However, of 4 relapsing stage II and 17 relapsing stage III patients, unfavorable histology was determined in 3 and 11 patients (missing data in 1 and 6 patients), respectively. Picard and colleagues reported no recurrences when the Ki67 index was <15%. In contrast, Ki67 index was <15% in 1 relapsing stage II and 1 stage III patient each in our study. In both studies, no events occurred when tumors were devoid of necrosis.

Several macroscopic tumor-related and microscopic features have been proposed as prognostic factors [1,4,7,8,11,16,17,18,19,20]. However, there is no single isolated feature that can differentiate between highly and less aggressive pediatric ACC and thus provide prognosis, as was the case in our study. The COG staging system includes major clinical (macroscopic) prognostic factors while microscopic pathological features were excluded [7,16]. Picard and colleagues included the most predictive microscopic criteria in the five-item score, thus incorporating elements that are independent of clinical features and were previously disregarded to guide management [11].

Adopted from an adult scoring system, a pediatric S-GRAS score has recently been proposed [14]. The pS-GRAS scoring system was evaluated in 733 patients including 126 and 95 ENSAT stage II and III patients, respectively. It considers the five categories T status, Ki67 or mitotic index, R status, age, and hormonal status and thus only one microscopic item. We did not observe significant differences between patients with pS-GRAS group 2, 3, and 4 for stage II and III patients. One of the key strengths of the pS-GRAS score is the combined evaluation of independent prognostic risk factors. By examining patients in separate tumor stages (one component of pS-GRAS), stage as a main risk factor loses its differentiating potential. As the data of Picard and us show, a single microscopic feature (in this case Ki67 or mitotic index) cannot distinguish between highly and less malignant tumors in specific subgroups.

Our study has several limitations. First, although large by international pediatric standards, the number of patients was small, particularly in the stage II COG subgroup, which made detailed analysis difficult and may have obscured differences. Due to the retrospective character of the study, we had to deal with data failure and dropouts. In the MET studies, treatment was recommended according to the TNM staging and not COG staging system. This may have influenced the outcome. We did not report other pathological criteria that were redundant with the five-item microscopic score, considering that two elements together in the same analysis for prognosis prediction are likely to overestimate malignancy. In the study by Picard and colleagues, pre-operative rupture determined by histopathological review showed a significant impact on progression-free survival. We did not gather these data.

Our data indicate that the five-item score in children and adolescents with COG stage III ACC identifies patients at high-risk of poor outcome. This confirms the EXPeRT/PARTNER recommendations [1]. It cannot confirm the prognostic value of the five-item score in stage II patients. However, this is most likely attributed to the small number of patients in this group. Collaborative efforts, including international prospective studies as well as molecular data, are needed to improve our understanding and management of patients with COG stage II tumors.

## 5. Conclusions

Pediatric ACC in COG stage II and III are the most unpredictable and challenging to prognosticate tumors and still have a dismal prognosis with a 3-year EFS of 76.5% and 49.8%, respectively. We demonstrated that patients in COG stage III with unfavorable histology had a worse outcome according to the five-item microscopy score, whereas no differences were observed in stage II patients and when subdivided into the pS-GRAS groups. Molecular studies are urgently needed to identify high-risk features in locally advanced tumors.

## Figures and Tables

**Table 1:** Patient characteristics of 18 children and adolescents with adrenocortical carcinoma COG stage II with and without event.

	COG Stage II with Event (*n* = 4)	COG Stage II without Event (*n* = 14)	*p* Value
Sex			1.000
Female	3 (75.0%)	11 (78.6%)	
Male	1 (25.0%)	3 (21.4%)	
Age at diagnosis (years)			0.047
Median, range	7.7 (1.6–17.8)	2.4 (0.8–11.2)	
Age groups			0.272
0–3 years	1 (9.1%)	10 (90.9%)	
≥4 years	3 (42.9%)	4 (57.1%)	
Stage of puberty			0.405
Prepubertal	2 (14.3%)	12 (85.7%)	
Pubertal	2 (50.0%)	2 (50.0%)	
Symptomatic interval prior to diagnosis (months)			0.230
Median, range	2.4 (0–4.8)	6.0 (1.2–30.0)	
Endocrine phenotype			0.170
Virilization	1 (25.0%)	10 (71.4%)	
Cushing syndrome	0	1 (7.1%)	
Combined	2 (50.0%)	1 (7.1%)	
Silent	1 (25.0%)	2 (14.3%)	
Laterality			0.641
Left	1 (25.0%)	6 (42.9%)	
Right	3 (75.0%)	7 (50.0%)	
Bilateral	0	1 (7.1%)	
T stage (according to AJCC system)			0.571
T1	0	0	
T2	3 (75.0%)	6 (46.2%)	
T3	0	1 (7.7%)	
T4	1 (25.0%)	6 (46.2%) *	
Tumor volume (ml)			0.491
Median, range	360.0 (125.8–904.8)	208.8 (114.8–3645.0)	
Five-item score			0.872
Favorable	0	3 (25.0%)	
Unfavorable	3 (100%)	9 (75.0%)	
pS-GRAS group			0.218
1	0	0	
2	0	5 (41.7%)	
3	2 (50.0%)	5 (41.7%)	
4	2 (50.0%)	2 (12.5%)	
Mitotane, adjuvant			1.000
No	3 (75.0)	12 (85.7%)	
Yes	1 (25.0)	2 (14.3%)	
Chemotherapy, adjuvant			1.000
No	3 (75.0)	11 (78.6%)	
Yes	1 (25.0)	3 (21.4%)	

* Frequencies were related to cases with recorded data.

**Table 2:** Patient characteristics of 37 children and adolescents with adrenocortical carcinoma COG stage III with and without event.

	COG Stage III with Event (*n* = 17)	COG Stage III without Event (*n* = 20)	*p* Value
Sex			0.992
Female	12 (70.6%)	13 (65.0%)	
Male	5 (29.4%)	7 (35.0%)	
Age at diagnosis (years)			0.968
Median, range	8.4 (1.9–17.1)	7.3 (0.3–16.0)	
Age groups			0.792
0–3 years	6 (40.0%)	9 (60.0%)	
≥4 years	11 (50.0%)	11 (50.0%)	
Stage of puberty			1.000
Prepubertal	10 (45.5%)	12 (54.5%)	
Pubertal	8 (53.3%)	7 (46.7%)	
Symptomatic interval prior to diagnosis (months)			0.548
Median, range	7.2 (1.2–32.4)	5.4 (0–91.2)	
Endocrine phenotype			0.692
Virilization	10 (66.7%)	10 (50.0%)	
Cushing syndrome	1 (6.7%)	3 (15.0%)	
Combined	3 (20.0%)	4 (20.0%)	
Silent	1 (6.7%) *	3 (15.0%)	
Laterality			0.648
Left	9 (52.9%)	8 (40.0%)	
Right	8 (47.1%)	12 (60.0%)	
T stage (according to AJCC system)			0.113
T1	0	2 (11.1)	
T2	12 (75.0%)	8 (44.4)	
T3	1 (6.3%)	0	
T4	3 (18.8%)	8 (44.4)	
Tumor volume (cm^3^)			0.017
Median, range	480.0 (30.0–2520.0)	136.7 (20.3–1254.0)	
N stage			0.441
N0	13 (81.3%)	19 (95.0%)	
N1	3 (18.8%)	1 (5.0%)	
Five-item score			0.017
Favorable	0	6 (54.5%)	
Unfavorable	11 (100%)	5 (45.5%)	
pS-GRAS group			0.819
1	0	0	
2	1 (7.1%)	2 (14.3%)	
3	5 (35.7%)	5 (35.7%)	
4	8 (57.1%)	7 (50.0%)	
Mitotane, adjuvant			1.000
No	9 (52.9)	9 (50.0)	
Yes	8 (47.1)	9 (50.0)	
Chemotherapy, adjuvant			1.000
No	8 (47.1)	8 (44.4)	
Yes	9 (52.9)	10 (55.6)	

* Frequencies were related to cases with recorded data.

## Data Availability

The data presented in this study are available upon reasonable request from the corresponding author. The data are not publicly available due to restrictions.

## Supplementary Materials

The following supporting information can be downloaded at: https://www.mdpi.com/article/10.3390/cancers15174296/s1, Figure S1: (a) Five-item microscopic score stratification according to Picard et al.; (b) pS-GRAS scoring system according to Riedmeier et al. [12].
