# Supplementary material for: Locally Advanced Adrenocortical Carcinoma in Children and Adolescents—Enigmatic and Challenging Cases
Source: Cancers (Basel). 2023 Aug 28;15(17):4296. doi: 10.3390/cancers15174296 (PMC10486626; doi:10.3390/cancers15174296)
Supplement: Supplementary file 1 [file cancers-15-04296-s001.zip › cancers-2560887-supplementary.pdf]

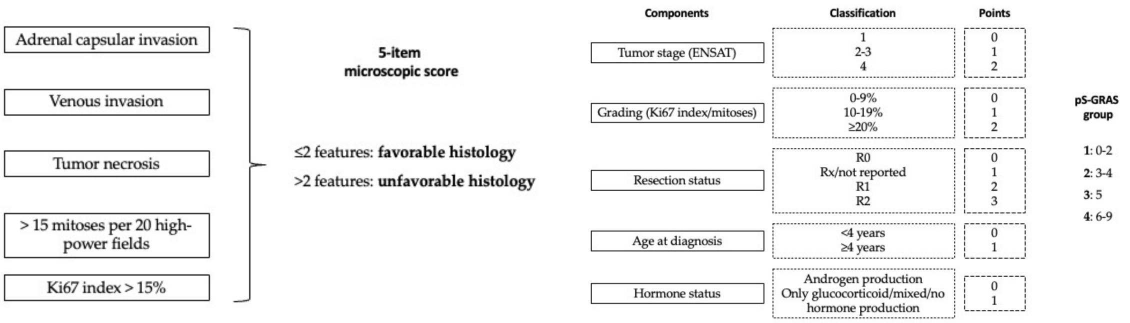

(**a**) (**b**)

Supplemental Figure S1. (**a**) Five-item microscopic score stratification according to Picard et al.; (**b**) pS-GRAS scoring system according to Riedmeier et al. [12].
